# A Data-Driven Customer-Search Modeling With the Consideration of Traffic Environment

**DOI:** 10.3389/fpubh.2022.848748

**Published:** 2022-03-17

**Authors:** Lan Yu, Zhuo Sun, Lianjie Jin, Chao Chen

**Affiliations:** ^1^College of Transportation Engineering, Dalian Maritime University, Dalian, China; ^2^State Key Laboratory of Structural Analysis of Industrial Equipment, School of Automotive Engineering, Dalian University of Technology, Dalian, China

**Keywords:** trajectory extraction, clustering algorithm, logit-based model, customer-search behavior, time-varying, en-route delay

## Abstract

In order to explore the determinants of vacant taxi drivers' customer-search behavior, this paper intends to calibrate a time-dependent Multinomial Logit (MNL) model by mining over 1.6 billion GPS records from about 8,400 taxis in Shanghai, China. First, based on the ordering points to identify the clustering structure (OPTICS) algorithm, the downtown area of Shanghai city is divided into 47 hotspots to identify the hot areas of customer delivery and searching. Then, by investigating a typical search delivery process of a vacant taxi, five candidate factors that may affect the customer-search behavior are summarized and defined. Using the maximum likelihood method, the significant factors are finally found. The results reveal that the relative passenger demand, the regional likelihood of pick-ups as well as the expected rate of return are the most significant factors influencing customer-search behavior. Although the impact of traffic situation (i.e., the en-route delay and traffic condition of the target hotspot) is not particularly significant, service providers and policymakers should still take full advantage of it to schedule taxi service and mitigate the traffic congestion caused by the circulation of vacant taxis. Besides, this paper also shows that the customer-search behavior of a vacant taxi driver varies with the time of day. Findings in this paper are expected to provide comprehensive insights about factors that should be considered in the future operation pattern of a taxi service system where human driver taxis and self-driving taxis are mixed.

## 1. Introduction

By providing demand-responsive, privacy, and flexible transport, taxi service plays a vital role in satisfying travel demand within an urban area. Compared with other public travel modes, taxis could offer a more comfortable and fast service and thus have been expanding their mode share of urban trips in recent years (1, 2). However, there are several tricky problems associated with the taxi service. First, during peak hours, it is difficult for a passenger to take a taxi in densely populated areas. The spatial-temporal mismatch between taxi demand and supply makes the service level of taxis in satisfying travel demand low.

Second, as taxis are an essential component of the traffic-flow in urban roads, the route choice behavior of taxi drivers has a significant effect on traffic conditions of the urban transportation network. Although the online taxi-hailing service has been growing sharply in recent years, most vacant taxi drivers still cannot accurately find a new passenger to serve after finishing an order. Based on past operational experiences, they usually circulate in the areas with high travel demand in search of passengers. The inefficient movement of vacant taxis could further increase traffic congestion and air pollution (3, 4).

In addition, conventional human-driven taxis are regarded as unsustainable due to the emission of carbon, invalid circulation, and the declining service level. By contrast, self-driving taxis, one of the hot topics in recent years, have been a suitable alternative. In the near future, the taxi service will be provided where human-driven taxis and self-driving taxis are mixed. Many potential benefits, such as the increase of service level and the improvement of urban traffic conditions, can be achieved if human-driven taxis work well with self-driving taxis. Therefore, understanding the customer-search behavior of human driver taxis is also critical to facilitating the deployment of self-driving taxis in the future taxi industry. Furthermore, it could also benefit ride-hailing types of taxis where people search a customer depending on their own experiences.

To tackle the issues involved in the taxi service, researchers have put forward various studies from different perspectives and practical methods. For the review of problems or models involved in the taxi service, interested readers could refer to Yang et al. (5) and Salanova et al. (6). The related works can be divided into three parts, 1) studies in regulatory policies, 2) studies in taxi network modeling, and 3) studies in customer-search behaviors.

Studies that relate to regulatory policies (7–10) often investigate the impact of the implementation of such policies, such as price control or entry restriction. However, with the assumption of the idealized taxi market, these studies were all analyzed from a macroscopic view. The spatial structure of the taxi market is not considered (11–14). In addition to such studies in regulator-aspect, many studies also paid attention to the taxi network modeling intending to capture the spatial structure of the taxi market (5, 15–19). It is worthy of note that the studies on taxi network modeling are mainly based on the assumption that taxi drivers search for passengers to minimize their searching time. However, this assumption indeed cannot reflect the real situation because the search time is not the only determinant affecting customer-search behavior. To better model the behavior of vacant taxi drivers, an increasing number of researchers paid great attention to finding factors that influence the customer-search behavior (11–14, 20–23).

The previous studies on search behaviors of vacant taxi drivers are mainly based on logit-based or probability-based form. Sirisoma et al. (11) conducted a stated preference survey of 400 taxi drivers in Hong Kong and developed a Multinomial Logit (MNL) model to analyze the choice mechanism. The results revealed that the trip time, toll, and waiting time are all significant factors affecting customer-search behavior. Besides, the demographic of drivers, such as age, marital status, driving experience, and vehicle ownership, also affected the choice mechanism. In recent years, the cost of GPS data collection has been deeply discounted with the rapid development of technologies and information systems. After collating and analyzing taxi GPS data, the route choices of taxi drivers in a real-world situation can be extracted, providing the essential data to understand the customer-search behavior of a vacant taxi driver.

Based on the GPS data of 460 taxis in Hong Kong, Szeto et al. (12) developed a time-depend logit-based model to study the customer-searching strategies of vacant taxi drivers over a day. Their results indicated that the passenger demand and rate of return are two significant factors that affect the searching behavior. Also, using the GPS data from 460 taxis in Hong Kong, Wong et al. (13) intended to seek the underlying mechanism of taxi customer-search behavior and validated a logit model, in which several factors were explored, including the cross-zonal travel distance, intra-zonal circulating distance, relative passenger demand and rate of return. To capture the effect of the cumulative probability of successfully picking passengers up in the customer-search process, Wong et al. (14) proposed a cell-based model combining the logit-based model and intervening opportunity model. In their paper, the GPS data of the 460 taxis in Hong Kong was also used to calibrate the model.

Apart from the theoretical works based on the theory of modeling, some empirical studies were also conducted to study the customer-search behavior of a vacant taxi driver. By using the continuous digital traces of more than 3,000 taxis in over 48 million trips, Liu et al. (20) made an empirical analysis of the operation patterns of taxi drivers. In their paper, the transportation congestion condition is a critical determinant for taxi drivers to search for customers. Veloso et al. (24) performed an exploratory analysis to understand the customer-search behavior in suburban areas by using the taxi-GPS traces collected in Lisbon, Portugal. They found taxi drivers prefer to search customers in areas with a higher probability of picking passengers up even if they need to travel a longer distance to that location. Through the taxi GPS data collected in Shenzhen, China, Zong et al. (23) developed a zero-inflated negative binomial model to identify the impacts of external and internal information (e.g., previous pick-up experience) on the cruising behaviors of taxi drivers. Their results indicated that external factors such as land use, traffic conditions, and road grade have a more significant influence than the internal ones (i.e., previous pick-up experience).

**Figure 1:**
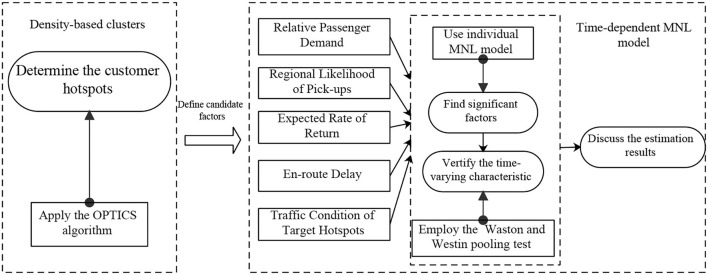
Overview of study methodology.

In summary, existing literature specifically focused on the study of customer-search behaviors has explored several factors affecting the search mechanism of vacant taxi driver, including search time/distance (13, 24, 25), revenue (20, 24), rate of return (13), passenger demand (13), pick-up likelihood (14, 24), land use (23), and transportation congestion condition (20). Though studies based on the modeling theory could provide the quantitative influence of those factors, they were still constrained by the small sample size. Quite the opposite, some empirical studies considered abundant factors in analyzing customer-search behavior; however, the significance of each factor cannot be quantified (20, 25). With the rapid development of vehicle-loaded GPS tracking devices, the available taxi data has been greatly enriched, providing the basis for deepening the understanding of customer-search behavior. In this paper, a data-driven taxi customer-search modeling is developed to fill the gap between the theoretical works and empirical studies on the customer-search behavior of a vacant taxi. Besides, to study the choice decisions of vacant taxi drivers, previous studies usually segmented the region into many small cells (14, 23, 24, 26) or different administrative districts (12, 20). To better reflect the demand distribution and search area preferences of vacant taxis, this paper utilizes the OPTICS algorithm to divide the study area into different customer hotspots. Furthermore, because of the temporal variations of passenger demand, the customer-search strategies of vacant taxi drivers indeed vary over the time of day (13, 14, 20, 23). To study the customer-search behavior of vacant taxi drivers in different periods, this paper develops and calibrates several time-dependent MNL models by mining over 1.6 billion GPS records from about 8400 taxis in Shanghai. Based on the Watson and Westin's pooling test (27), the time-varying search strategies are finally investigated and approved. An overview of the study methodology is also presented in Figure 1.

The contributions of this paper lie in the following.

1. Using the OPTICS algorithm, the study area of this paper is segmented into different customer hotspots. Unlike previous studies that usually divided a mega-region into many small cells or administrative districts, this method could better reflect taxi drivers' demand distribution and searching area preferences.
2. By investigating a typical search delivery process of a vacant taxi, five candidate factors that may have an influence on the customer-search behavior, including the relative passenger demand, regional likelihood of pick-ups, expected rate of return, en-route delay, as well as the traffic condition of target hotspot are defined.
3. Several time-dependent MNL models are developed to provide empirical evidence about the significant factors in the customer-search mechanism of a vacant taxi driver. Besides, the Waston and Westin pooling test is also conducted in this paper to reveal the time-varying characteristic of customer-search behavior.

The rest of this paper is structured as follows. Section 2 describes the raw data, initial data processing algorithms, as well as the study area and time. Section 3 presents the methodology on the determination of customer hotspots and logit-based taxi customer-search behavior model. Section 4 provides the detailed results. Finally, concluding remarks and outlooks for future work are drawn in Section 5.

## 2. Data Preparation

### 2.1. Data Acquisition

Taxi GPS data used in this paper is collected from the Shanghai Qiangsheng Taxi Company. The number of taxis operated by Shanghai Qiangsheng Taxi Company occupies approximately 30% of the entire Shanghai taxi population, and it can adequately represent the behaviors of taxi drivers in Shanghai (3). The raw data collected every 10s for over a month (March 2016) contain about 8400 taxis and more than 1.6 billion GPS records in total. The database records the location in terms of longitude and latitude, vehicle identification, spot speed (km/h), timestamp, and operation status (empty or occupied). We should mention that the data includes both occupied and empty running trips and could describe the full operation behaviors of taxi drivers.

### 2.2. Data Clean, Processing, and Extraction

To obtain the pick-up and drop-off points, as well as those occupied and empty trips, some initial data processing procedures are firstly carried out. These procedures include data cleaning, coordinate transformation, and map matching. Then an Origin-Destination (OD) extraction and mapping algorithm based on time series are designed. This algorithm provides the data foundation for this paper. The trip distance, travel time, and other essential parameters thus can be ultimately calculated.

In this paper, the pick-up point can be considered as a point where the state of the taxi is translated from empty to occupied. Similarly, the drop-off point is a transition of the state of a taxi from occupied to empty. Thus, based on the status change, the procedure of the OD extraction and mapping algorithm can be designed and is shown in Appendix A.

### 2.3. Travel Fare and Operational Cost

To develop the MNL model, the taxi fare and operational cost should also be mentioned. The fare structure adopted in Shanghai is based on a non-linear fare structure. In the day-time, the initial charge for the first 3 km is 13 CNY and an additional 2.4 CNY charge for every subsequent 1 km or every 5 min waiting time. Trips after 11 pm and before 5 am will start at 16 CNY and will be charged an additional 3.1 CNY compared to the day-time 2.4 CNY. Besides, passengers also must pay 1 CNY for the fuel surcharge in both day-time and mid-night.

Before introducing the operational cost, it is necessary to describe the work-pattern of taxi drivers. In Shanghai, most taxis run 24h a day but in two shifts: The day shift is from 4 am to 4 pm, and the night shift is from 4 pm to 4 am. Thus, each vehicle has two drivers. Based on the report provided by the Shanghai Qiangsheng Taxi Company, the monthly taxi rental cost in 2016 for a single driver is 4,700 CNY. With the shift of 12h and the operating days of 30 days, the rental cost can be calculated as 0.22 CNY/min.

Apart from the rental cost, the fuel cost also accounts for a large proportion of the total operational cost of taxis. In 2016, all taxis of Shanghai Qiangsheng Taxi Company were running on petrol. Based on a survey of taxi drivers, the fuel consumption of a regular taxi is approximately 8 to 10 liters per 100 kilometers. Therefore, the fuel consumption of taxis per 100 kilometers in this paper is set as 9 liters. The unit price of petrol in 2016, Shanghai, is 6.87 CNY/liter. Thus, the unit fuel cost of taxis is 0.6 CNY/km. Although there are no data on precise fare collection, the approximate fare can be calculated by the vehicle trajectory (trip distance and travel time) deduced by the taxi location and speed information. The approximate operational cost can be calculated in the same way.

### 2.4. Study Region and Time

**Figure 2:**
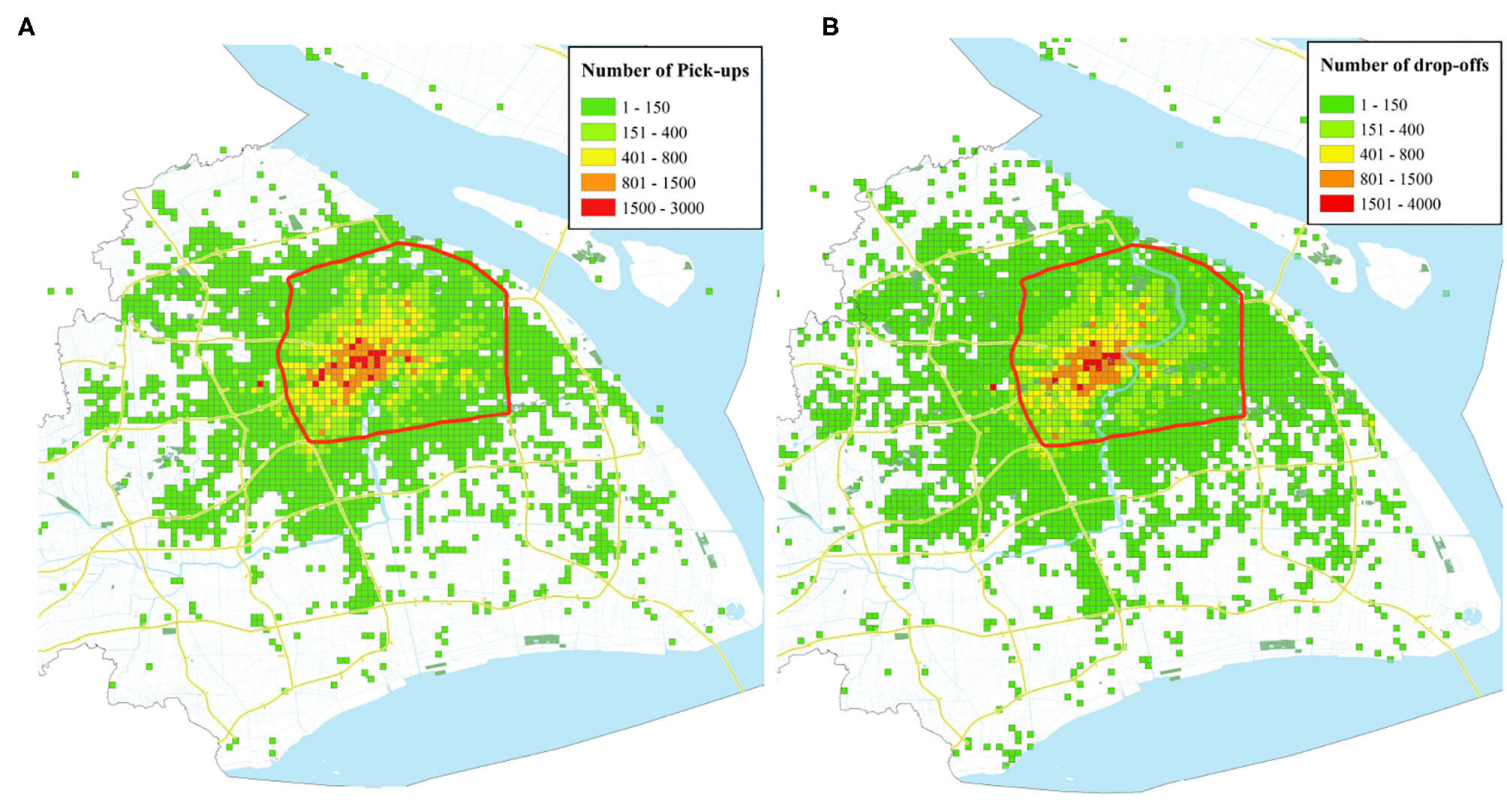
The study area and demand distribution during a typical workday. **(A)** The distribution of pick-ups. **(B)** The distribution of drop-offs.

As shown in Figure 2, the distribution of pick-ups and drop-offs of taxis from QiangSheng Company during a typical workday covers almost the entire city of Shanghai. Furthermore, the Inner Ring Road, denoted by the red circle, encompasses the downtown area of Shanghai. The pick-ups and drop-offs within the Inner Ring Road contribute approximately up to 89.85 and 87.89% of daily pick-ups and drop-offs, respectively. This area can be regarded as the active service region of taxis. Thus, in this paper, the area of Shanghai within the Inner Ring Road is selected as the study area.

**Figure 3:**
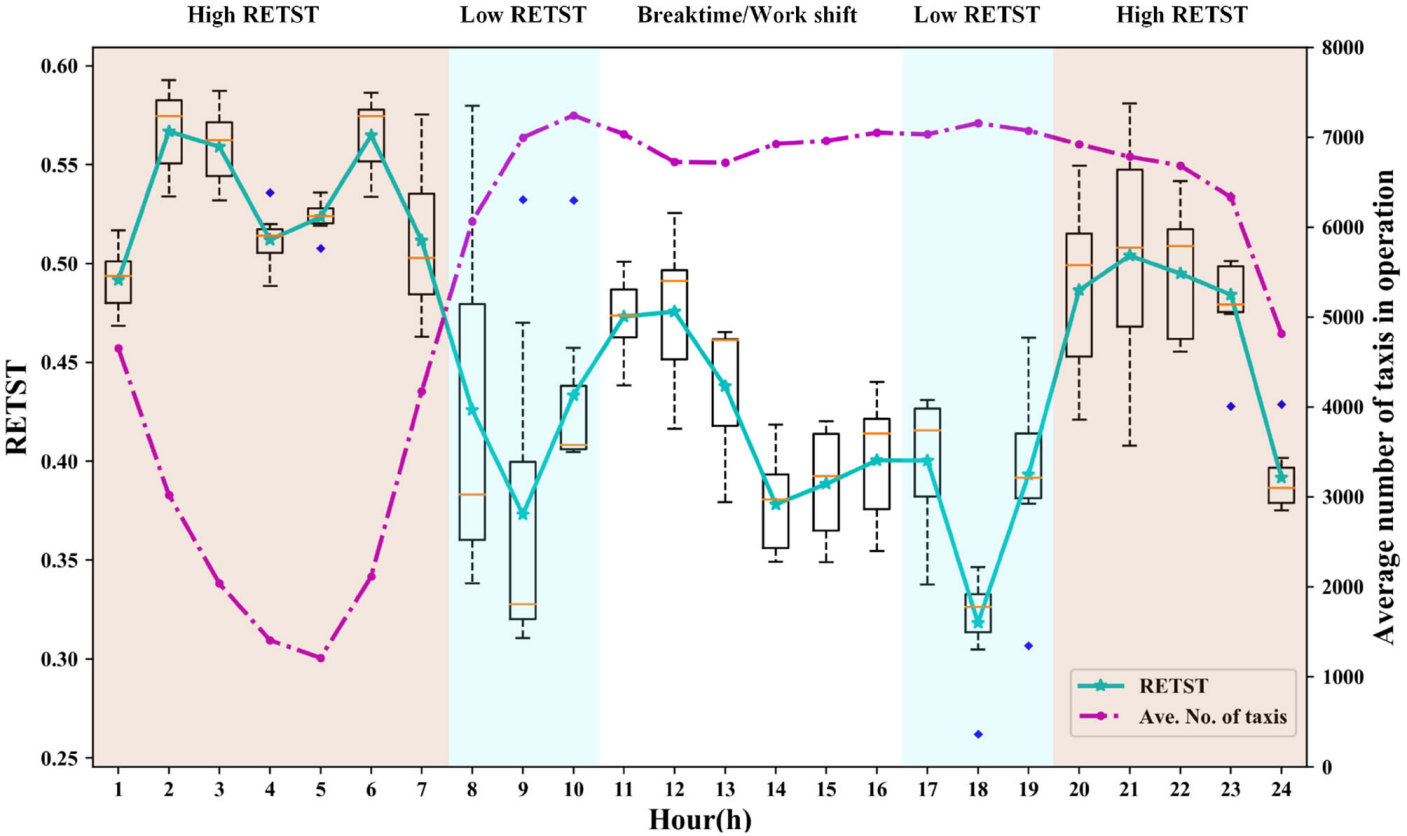
Profiles of the ratio of empty/total service time (RETST) and the average number of taxis in operation.

The customer-search behavior of vacant taxis indeed varies with time (12). It depends on the balance of the demand and taxis in operation (i.e., the level of competition). To accurately find factors affecting customer-search behavior, it is necessary to divide the time into several spans. In this paper, the ratio of empty/total service time (RETST) is adopted to show the level of competition. Figure 3 shows the trends of the RETST and the average number of taxis in operation over a typical day in March 2016.

The low RETST during peak hours (7:00–10:00 and 16:00–19:00) means that the supply falls nearly short of demand. During this period, vacant taxi drivers could quickly get new orders. By contrast, the occurrence of high RETST after the evening rush hour and before dawn (23:00–7:00) denotes that the supply exceeds the demand. During this period, taxi drivers prefer not to provide service, and the number of taxis in operation reaches the minimum at 5 a.m. It should also be noted that the average number of taxis in operation falls slightly from 10:00 to 13:00 and then increases from 13:00 to 16:00. That may be attributed to that in Shanghai, taxi drivers usually have a flexible meal schedule and shift change during that time. However, it is difficult to distinguish betweenthe status of taking a meal, having a shift change, or waiting for new orders. To avoid the bothers brought by the small sample size, break-time, and work shift, we omit data collected during 23:00–7:00 and 10:00–16:00 and eventually select the continuous periods 7:00–10:00, 17:00–19:00, and 20:00–23:00 as our study time.

Thus, the customer-search behaviors of vacant taxi drivers during peak period (i.e., 7:00–10:00 and 17:00–19:00) and off-peak period (i.e., 20:00–23:00) are studied, respectively.

## 3. Methodology

### 3.1. Determination of Customer Hotspots

In order to study the customer-search behavior of a vacant taxi driver from a microcosmic perspective, it is necessary to separate the whole city area into many relatively small ones. Previous studies usually segmented the study region into small cells (14, 26, 28, 29) or different administrative districts (12, 14, 20, 30). However, these subdivision methods can not reflect the demand distribution and searching area preferences of vacant taxis. Unlike previous studies, in this paper, to alleviate the burden of data processing, the study area (i.e., the Shanghai city) is divided into different customer hotspots based on the OPTICS algorithm. The hotspot in this paper refers to a specific region where vacant taxis prefer to search for customers.

The OPTICS algorithm, first proposed by Ankerst et al. (31), is one of the most popular density-based clustering methods. The principle of the OPTICS is equivalent to that of an extended density-based spatial clustering of application with noise (DBSCAN) algorithm. Similarly, the OPTIC algorithm depends on the three parameters defined in the DBSCAN algorithm, including the generation distance (*d*), the radius of cluster (ε), and the minimum number of points required for a cluster (*MinPts*). However, unlike the DBSCAN explicitly generating the clusters for data set, this method produces a density-based clustering structure of the data in the order of the points. Besides, the OPTIC can also avoid the trouble of parameter selection in the DBSCAN algorithm (31, 32). It should be mentioned that a set of information, including the core distance (*cd*) and the reachability distance (*rd*), is stored in the density-based cluster structure. This kind of information can be finally utilized to extract the clusters and identify noises. For more details of the OPTICS algorithm, interested readers could refer to Ankerst et al. (31).

Based on the concepts of the OPTICS algorithm discussed above, the procedure of the clustering algorithm for our problem is designed. However, before giving the pseudo-code of the OPTICS algorithm, related symbols and functions should be first introduced. *I* is the data set, *N*_ε_(*i*) is the ε-neighborhood of data point *i*, *c*_*i*_ and *r*_*i*_ are the core distance and reachability distance of data point *i*, respectively. *v*_*i*_ is the sign variable indicating whether data point *i* is traversed or not. *Seedlist* is the temporary set of data points that have been processed and ordered by the reachability distance in ascending. *P* is the sequence of all data points in ascending order of reachability distance, and *M* is the clustering result. Then, the procedure of the OPTICS is shown in the following.

The function *insertlist*() is indeed a sub-procedure of the Algorithm 1. It is used to update the reachability distance of the data that have been processed. This function is repeated called by the OPTICS algorithm until all data points are traversed, and the pseudo-code is shown as follows.

**Algorithm 1:** OPTICS algorithm.

1: //*Initialization*//
2: Given parameter ε, *MinPts*
3: Generate *N*_ε_(*i*) and *c*_*i*_, *i* = 1, 2..., *N*
4: Set *k* = 1, *v*_*i*_ = 0, *r*_*i*_ = *UNDEFINED*, *i* = 1, 2..., *N*
5: Set *I* = {1, 2, ..., *N*} and *seedlist* = ∅
6: //*OPTICS*//
7: **While** *I* ≠ ∅ **do**
8: Get an element *i* ∈ *I* and set *I* = *I*\{*i*}
9: **If** *v*_*i*_ = 0 **then**
10: Set *v*_*i*_ = 1, *p*_*k*_ = *i*, and *k* = *k* + 1
11: **If** |*N*_ε_(*i*)|≥*MinPts* **then**
12: *insertlist* $\left(N_{\varepsilon }\left(i\right),{\left\{v_{l}\right\}}_{l=1}^{N},{\left\{r_{l}\right\}}_{l=1}^{N},c_{i},seedlist\right)$
13: **While** *seedlist* ≠ ∅ **do**
14: Get the first element *j* ∈ *seedlist*
15: Set *v*_*j*_ = 1, *p*_*k*_ = *j*, and *k* = *k* + 1
16: **If** |*N*_ε_(*j*)|≥*MinPts* **then**
17: *insertlist* $\left(N_{\varepsilon }\left(j\right),{\left\{v_{l}\right\}}_{l=1}^{N},{\left\{r_{l}\right\}}_{l=1}^{N},c_{j},seedlist\right)$
18: **EndIf**
19: **EndWhile**
20: **EndIf**
21: **EndIf**
22: **EndWhile**
23: Output $P={\left\{P_{i}\right\}}_{i=1}^{N}$
24: //*OPTICSClusteringExtracting*//
25: Given parameter $\tilde{\varepsilon }$ $\left(\tilde{\varepsilon }\leq \varepsilon \right)$
26: Set *clusterID* = −1, and *n* = 1
27: **For** *i* = 1, 2, ..., *N* **do**
28: *t* = *p*_*i*_
29: **If** $r_{t}>\tilde{\varepsilon }$ or *r*_*t*_ = *UNDEFINED* **then**
30: **If** *c*_*t*_ ≠ *UNDEFINED* and $c_{t}\leq \tilde{\varepsilon }$ **then**
31: *clusterID* = *n*
32: *n* = *n* + 1
33: *m*_*t*_ = *clusterID*
34: **Else**
35: *m*_*t*_ = −1
36: **EndIf**
37: **Else**
38: *m*_*t*_ = *clusterID*
39: **EndIf**
40: **EndFor**
41: Output $M={\left\{m_{i}\right\}}_{i=1}^{N}$

**Algorithm 2:** The procedure of *insertlist*().

1: **For** *h* ∈ *N*_ε_(*H*) **do**
2: **If** *v*_*h*_ = 0 **then**
3: rd=*max*{*cd*_*H, d*(*x*^(*H*)^, *x*^(*h*)^)}
4: **If** *r*_*h*_ = *UNDEFINED* **then**
5: Set *r*_*h*_ = *rd*
6: Insert *h* into *seedlist* and sort *seedlist* in accending order
7: **Else**
8: **If** *rd*< *r*_*h*_ **then**
9: Set *r*_*h*_ = *rd*
10: Insert *h* into *seedlist* and sort *seedlist* in accending order
11: **EndIf**
12: **EndIf**
13: **EndIf**
14: **EndFor**

Thus, the study of vacant taxi customer-search behavior in this paper turns into the analysis of the customer hotspot choice decision. Based on the historical taxi trajectory data and the algorithm, the spatial characteristics of pick-ups and drop-offs can also be explored.

### 3.2. Candidate Factors Affecting the Vacant Taxi Customer-Search Behavior

**Figure 4:**
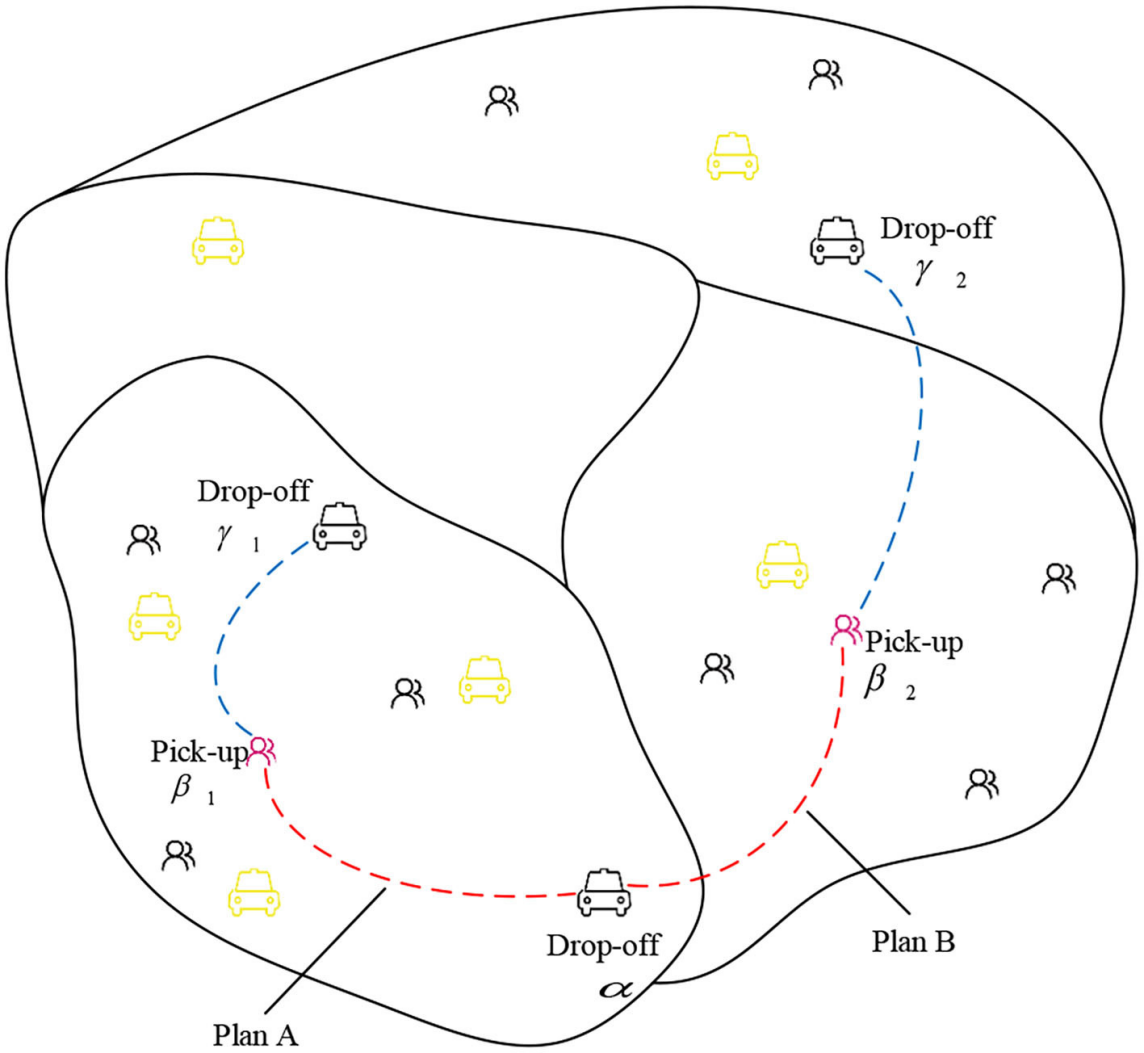
A typical customer search and delivery process.

Vacant taxi customer-search is a high-level human behavior and complex decision-making process that incorporates the drivers' past and unspoken experiences. Previous studies have explored several factors that may influence the vacant taxi customer-search behavior (11, 12). Concerning these factors, the en-route delay and the traffic condition of the target hotspot are always ignored. To find factors affecting customer-search behavior comprehensively, the overall customer-search and customer-delivery process should be studied. A typical process of customer search and delivery is shown in Figure 4.

As illustrated in Figure 4, a studied taxi finishes the current order at drop-off location α and is going to search for the next passenger. The demand in the target area influences the customer-search decision. Due to the high demand, searching for a customer successfully in a short period becomes promising. In other words, relative passenger demand is an essential factor that may affect customer-search behavior. However, in the search process (α → β), high passenger demand cannot ensure the probability of successfully picking passengers up. Vacant taxi drivers prefer to search through an area, in which passenger demand is great, and quantity supplied (i.e., the number of vacant taxis) is small. The probability of a vacant taxi driver successfully picking a passenger up decreases if there are many vacant taxis nearby. Thus, the regional likelihood of pick-ups is also a critical candidate factor. The traffic condition along the way of searching customers is also likely to affect customer-search behavior. The low speed caused by traffic jams will deter vacant taxi drivers from searching for passengers. Similarly, the traffic condition of the target area also needs to be considered. Finally, the fare, travel expense, and time (i.e., the expected rate of return) should also be considered because vacant taxi drivers prefer passengers with high travel fare and low searching time and cost.

In this paper, the vacant taxi customer-search behavior is considered as the choice to select a particular customer hotspot in the city region. The choice can either be intra-hotspot circulating or cross-zonal traveling. Besides, these choices are assumed to be independent. By analyzing the typical search and delivery process of a vacant taxi driver, five factors that may have an effect on the customer-search behavior are summarized and defined. The associated definitions and formulates for the five factors, including the relative passenger demand, regional likelihood of pick-ups, expected rate of return, en-route delay, as well as traffic condition of target hotspot, are presented in the following.

#### 3.2.1. The Relative Passenger Demand

Passenger demand is the most important factor considered by vacant taxi drivers in finding customers. They always prefer to cruise in areas with higher passenger demand according to their experience. In this paper, the relative passenger demand of hotspot (*i*) is used to capture the attractiveness of high demand, and the definition is shown as follows.

**Definition 1**. Given the number of passengers picked up in hotspot *i* at time interval *t*, $P_{i}^{t}$, and the total number of occupied trips in the entire study area, the relative passenger demand $E_{i}^{t}$ is defined as follows.


(1) $$\begin{array}{l} E_{i}^{t}=\frac{P_{i}^{t}}{\sum_{i\in N}P_{i}^{t}} \end{array}$$

#### 3.2.2. The Regional Likelihood of Pick-Ups

Higher passenger demand in one area cannot ensure a reasonable probability of successfully picking passengers up. It can be offset by the heavy competition. Therefore, the regional likelihood of pick-ups should also be a factor that needs to be considered.

**Definition 2**. Given the total number of vacant taxi $V_{i}^{t}$ and occupied taxi $O_{i}^{t}$ in hotspot *i* at time interval *t*, the regional likelihood of pick-ups, $LP_{i}^{t}$, can be formulated as:


(2) $$\begin{array}{l} LP_{i}^{t}=\frac{V_{i}^{t}}{V_{i}^{t}+O_{i}^{t}} \end{array}$$

#### 3.2.3. Expected Rate of Return

When searching for the next customer, the vacant taxi driver will consider not only the time and cost consumed from the current location to the next pick-up location but also the expected profit brought by the next order, which will eventually affect their incomes. Thus, the expected rate of return is a candidate factor to be considered. Before calculating the expected rate of return, three related definitions, including the travel time, travel expense, and fare, are first given below.

**Definition 3**. The total travel time of trip *k* of an individual vacant taxi driver starting from hotspot *i* and aiming *j* to search for the next customer $T_{k,i,j}^{t}$.


(3) $$\begin{array}{l} T_{k,i,j}^{t}=ST_{k,i,j}^{t}+DT_{k,i,j}^{t} \end{array}$$

where $ST_{k,i,j}^{t}$ is the searching time of trip *k* of an individual vacant taxi driver from hotspot *i* to *j* at time interval *t*; and $DT_{k,i,j}^{t}$ is the service time (i.e. delivery time) of trip *k*.

**Definition 4**. For a typical customer search and delivery process, the travel expense for a vacant taxi driver is comprised of two parts: one is the rental cost, and the other is the fuel cost. Thus, the travel expense of trip *k* of an individual vacant taxi driver from hotspot *i* to *j* at time interval *t*, $C_{k,i,j}^{t}$, can be calculated as follows.


(4) $$\begin{array}{l} C_{k,i,j}^{t}=C^{r}\cdot T_{k,i,j}^{t}+C^{f}\cdot \left(SD_{k,i,j}^{t}+DD_{k,i,j}^{t}\right) \end{array}$$

where *C*^*r*^ is the rental cost per unit of time; *C*^*f*^ is the fuel cost per unit of distance; $SD_{k,i,j}^{t}$ is the search distance of trip *k* in customer-search process from hotspot *i* to *j*; $DD_{k,i,j}^{t}$ is the delivery distance of the occupied trip *k* from hotspot *j*.

**Definition 5**. The fare of trip *k* of an individual vacant taxi driver from hotspot *i* to *j* at time interval *t* can be calculated based on time interval, fixed fuel surcharge fare, and the delivery distance, which is formulated as follows.


(5) $$\begin{array}{l} F_{k,i,j}^{t}=Fc+F\left(s\right) \end{array}$$

Where *Fc* is the fixed fuel surcharge fare, *s* denotes the delivery cost, and *F*(·) is the function of travel fare calculation discussed in Section 2.3.

We can, therefore, define, hereafter, the expected rate of return as follows.

**Definition 6**. The expected rate of return (EROR) is calculated based on travel fare, expense, travel time, and the time interval, which is denoted as follows.


(6) $$\begin{array}{l} R_{k,i,j}^{t}=\frac{F_{k,i,j}^{t}-C_{k,i,j}^{t}}{T_{k,i,j}^{t}} \end{array}$$

The numerator of the Equation 6 shows the expected profit of trip *k* of an individual vacant taxi driver from hotspot *i* to *j* at time interval *t*. Furthermore, the denominator represents the expected time taken to obtain that profit. The two numbers are calculated based on an intact customer search and delivery process. The value of the ratio describes the effect of perceived profit in the decision-making process when vacant taxi drivers search for the next customer. A higher EROR may result in a higher probability of a vacant taxi driver going from one hotspot to the designated hotspot.

#### 3.2.4. En-route Delay

The en-route delay reflects the traffic condition along the way of searching for customers. With the same searching distance, severe en-route delay may increase the searching time and cost, even destroying the light mood. Thus, it should be one another candidate factor affecting the customer-search behavior of a vacant taxi driver. En-route delay is indeed correlated with several factors, such as type of road, road capacity, and traffic accident. However, it is challenging to incorporate all factors into one parameter to represent the en-route delay. In practice, experienced drivers are more likely to search passengers through a route with a stable travel speed. Thus, in this paper, the en-route delay is represented by the standard deviation of customer-search time between the current hotspot and the designated hotspot, and the definition is given below. Note that the en-route delay in this paper does not intend to represent traffic conditions of a specific road. Instead, it aims to reflect the general road traffic conditions between two hotspots partly.

**Definition 7**. Given the average customer-search time, and the total number of search trips (i.e., empty trips) from hotspot *i* to *j*, the en-route delay can be mathematically expressed as follows.


(7) $$\begin{array}{l} ED_{i,j}^{t}=\sqrt{\frac{1}{N_{i,j}-1}\underset{k}{\sum }{\left(ST_{k,i,j}^{t}-{\bar{ST}}_{i,j}^{t}\right)}^{2}} \end{array}$$

Where *N*_*ij*_ indicates the total number of search trips from hotspot *i* to *j*. $ST_{k,i,j}^{t}$ is the search time of trip *k* of an individual vacant taxi driver from hotspot *i* to *j* at time interval *t*. ${\bar{ST}}_{i,j}^{t}$ is the average customer-search time from hotspot *i* to *j* at time interval *t*, which is calculated as $\underset{k}{\sum }\frac{ST_{k,i,j}^{t}}{N_{i,j}}$.

#### 3.2.5. Traffic Condition of Target Region

Similarly, the traffic condition of target hotspot *j* will also affect the vacant taxi customer-search behavior. A congested traffic condition may cause taxi drivers to move slowly and consume more time to deliver the passengers. In this paper, the average speed is adopted to reflect the traffic condition of the target region *j*.

**Definition 8**. The average speed of target region *j* at time interval *t*, $V_{j}^{t}$, is a ratio of search and delivery distance to the search and delivery time.


(8) $$\begin{array}{l} {\bar{V}}_{j}^{t}=\frac{\sum_{k}\left(SD_{k,j}^{t}+DD_{k,j}^{t}\right)}{\sum_{k}\left(ST_{k,j}^{t}+DT_{k,j}^{t}\right)} \end{array}$$

where $SD_{k,j}^{t}$ is the search distance before trip *k*. In contrast, $DD_{k,j}^{t}$ is the delivery distance of trip *k* starting from hotspot *j* at time interval *t*. And $ST_{k,j}^{t}$ and $DT_{k,j}^{t}$ denote the corresponding search time and delivery time, respectively.

For every single search-delivery trip, the five factors are calculated using the data introduced in Section 2. Then 22,507 samples are drawn for the off-peak period, 25,923 samples are drawn for the morning peak hours, and 16,864 samples for the evening peak hours.

### 3.3. Multinomial Logit Model for Customer-Search Behavior

In this paper, the MNL model is employed to analyze the customer-search behavior of a vacant taxi driver. Based on the theory of discrete choice, a vacant taxi driver chooses the designated hotspot, which brings him the highest utility. The utility (*U*_*ijk*_) when the vacant taxi driver is currently in hotspot *i* and about to go to hotspot *j* for searching the next passenger in trip *k*, consists of two parts: measurable utility (*V*_*ijk*_) and unmeasured utility (ε_*ijk*_):


(9) $$\begin{array}{l} U_{ijk}=V_{ijk}+\varepsilon_{ijk} \end{array}$$

Based on the candidate factors presented in Section 3.2, the measurable utility function can be mathematically expressed as follows.


(10) $$\begin{array}{l} V_{ijk}=\delta_{j}+\beta^{E}E_{j}^{t}+\beta^{LP}LP_{j}^{t}+\beta^{R}R_{k,i,j}^{t}+\beta^{ED}ED_{ij}^{t}+\beta^{\bar{V}}\bar{V_{j}^{t}} \end{array}$$

Where, δ_*j*_ is the utility constant of hotspot *j*; β^*E*^ is the coefficient associated with the relative passenger demand; β^*LP*^ is the coefficient associated with the regional likelihood of pick-ups; β^*R*^ is the coefficient associated with the expected rate of return; β^*ED*^ is the coefficient associated with the en-route delay and $\beta^{\bar{V}}$ is the coefficient associated with the traffic condition of target hotspot.

When the factors of measurable utility and the unmeasured utility are independent, and the random utility (ε_*ijk*_) is the unobserved error component obeying Gumbel distribution, the probability that the vacant taxi driver currently in hotspot *i* chooses hotspot *j* to start trip *k* can be obtained by the following form.


(11) $$\begin{array}{l} P_{k}\left(j|i\right)=\frac{e^{V^{ijk}}}{\sum_{j}e^{V_{ijk}}},i=1,2,...,N;j\in K_{i} \end{array}$$

Where, *K*_*i*_ is the set of optional hotspots for the vacant taxi located in hotspot *i*.

### 3.4. Multicollinearity Detection

As the classical linear regression, the multicollinearity problem is also involved in the MNL model. It affects the stability of models and may cause the incorrect identification of influence factors (33). In this paper, two methods, Pearson product-moment correlation coefficient (PPMCC) and the variance inflation factor (VIF), are introduced to detect the presence of multicollinearity. The former method is to identify the linear correlation between any two variables in the five-candidate factors. Variables with the coefficients (absolute values) higher than 0.7 are considered to have the multicollinearity and will be removed in the MNL model. The latter method is used to reduce the likelihood of any multicollinearity further. VIFs equal to 1 indicates that there is no collinearity existing in the variables of the model. By contrast, VIFs larger than 10 present the severe collinearity (34). Thus, in this paper, variables with VIFs higher than 10 will also be eliminated in the model.

### 3.5. Watson and Westin Pooling Test

The customer-search behavior of a vacant taxi driver appears not to be constant for different periods (12, 13, 20, 26). Taxi drivers will circulate the city area in different patterns in 1 day to maximize their profits. Thus, it is necessary to understand the underlying behavior patterns for different periods to mitigate the traffic congestion caused by the unnecessary circulation of vacant taxis and improve the income of taxi drivers. In this paper, four combined MNL models with different periods were first developed and listed in Table 1.

**Table 1:** Combined MNL models with different time periods.

**Model**	**Time periods**		
	**Morning-peak hours**	**Off-peak hours**	**Evening-peak hours**
1	√	√	
2	√		√
3		√	√
4	√	√	√

Then the Watson and Westin pooling test approach was applied to study the sensitivity of the periods (12, 13, 27, 35, 36). This method is based on the log-likelihood ratio, and the associated equation is shown as follows.


(12) $$\begin{array}{l} LR=-2\left(L_{R}-L_{U}\right) \end{array}$$

Where *L*_*R*_ is the log-likelihood of the combined MNL model with two or three different periods, and *L*_*U*_ is the sum of the log-likelihoods of the individual MNL models developed for different periods. The null hypothesis of the test approach is that there is no difference between the individual MNL model and the combined MNL model. If the test statistic is larger than the threshold value (i.e., the value specified by the chi-squared distribution at a chosen level of significance), the null hypothesis is rejected. Besides, the degree of freedom can be calculated by subtracting the number of variables of the combined MNL models from the sum of the number of individual MNL models.

## 4. Results and Discussion

### 4.1. Characteristics of Trips and Candidate Factors

**Figure 5:**
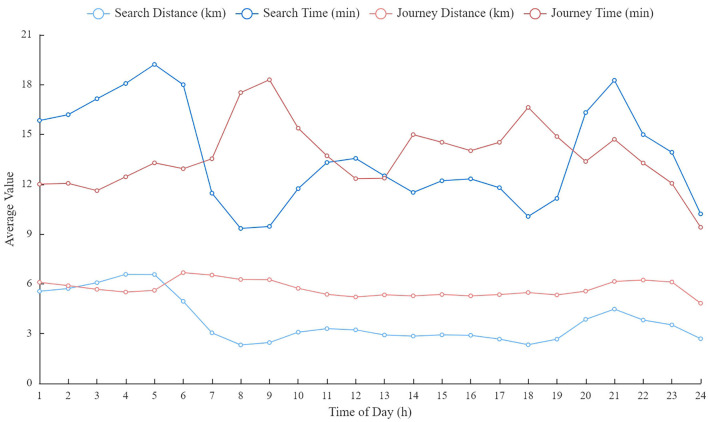
The distribution of trip characteristics on the time of a typical day.

Figure 5 shows the distribution of trip characteristics at different times of a typical day. In the morning (or evening) peak hours (7:00–10:00 or 17:00–19:00), vacant taxi drivers spent less time and traveled less distance to find the next customers than off-peak hours due to the high demand. By contrast, the search distance in the midnight period, on average, triples during peak hours. This demonstrates that vacant taxi drivers need to travel more distance to find their next customer in midnight period. Additionally, the journey distances present no appreciable difference in different periods, which is inconsistent with findings in previous research (13). Even more adverse, the journey distance in morning peak hours is higher than other hours. It is potentially related to the spatial division of residential and working areas in Shanghai City. However, in off-peak and mid-night periods, the journal time is notably short than peak hours. It can be attributed to the relatively good traffic condition, enabling taxi drivers to have a higher traffic speed. More details of the trip characteristics can be found in Appendix B.

Based on the analysis in Figure 5, the taxi market shows a significant fluctuation across a day. The vacant taxi drivers may use different customer-search strategies to increase their incomes. Therefore, the customer-search behaviors of vacant taxi drivers in different periods are studied separately. The behavior-changing across the day will also be explored.

### 4.2. Clustering Results Based on OPTICS

As discussed, the hotspot refers to a specific region where vacant taxis prefer to search for customers. In this paper, these hotspots are identified using the data of pick-ups in peak hours (including morning and evening peak hours). Note that the commonly used method in the OPTICS algorithm to identify clusterings is to utilize the reachability plot, a scatter diagram showing the reachability distance of each point in the ascending order. Using this plot, the hierarchical structure of the clusters can be easily obtained. In this figure, the x-axis represents the ordering of the points processed by OPTICS, while the y-axis represents the reachability distance.

**Figure 6:**
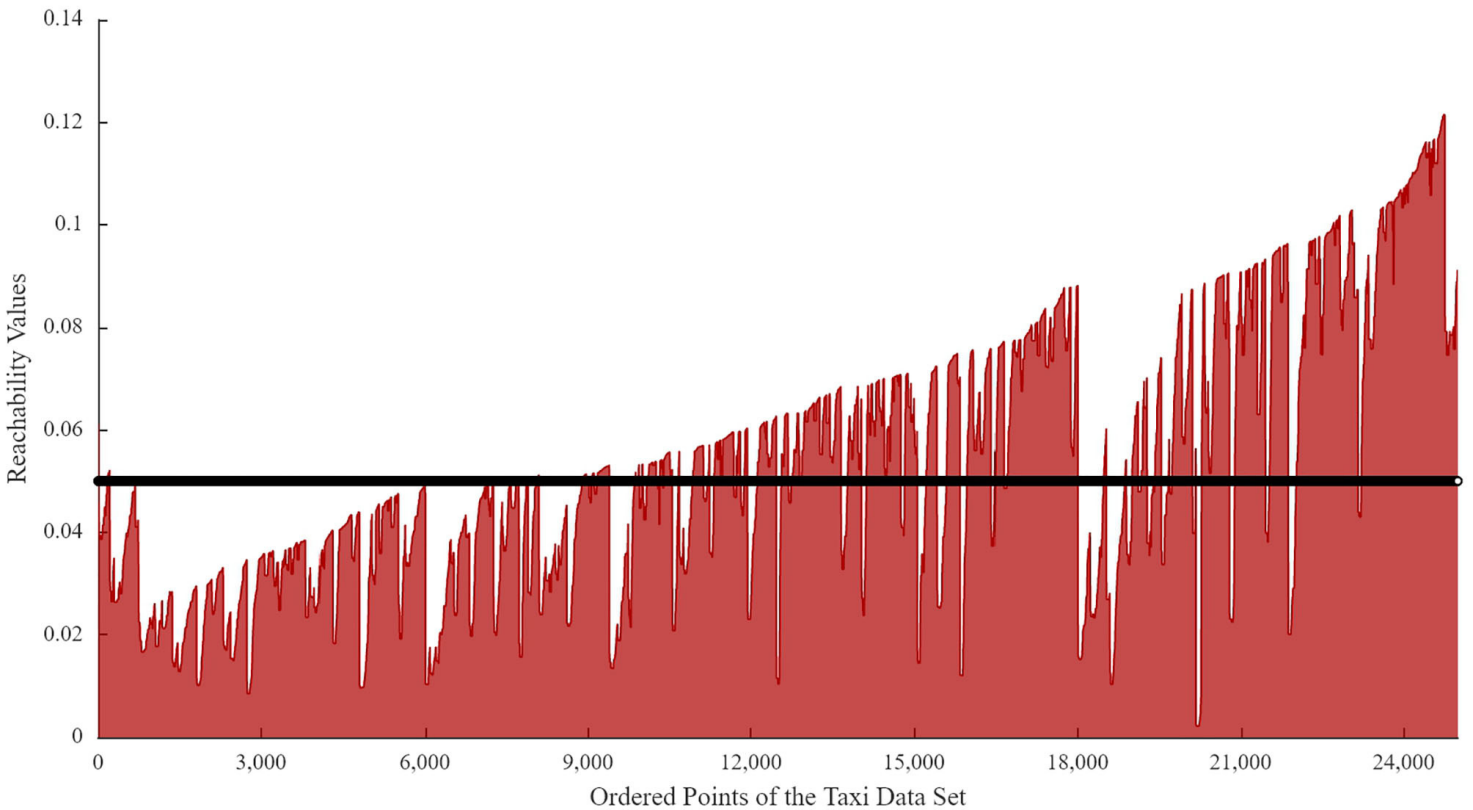
The reachability plot for the data set of pick-ups in peak hours.

To determine the best parameters, the range of the two parameters is set as *MinPts* ∈ {20, 30, 40, 50, 60, 70, 80, 90} and ε ∈ [0.01, 0.06]. Then, a grid search is performed. Results indicate that the value of the parameter, *MinPts*, is 50. Given the value of *MinPts* is 50, the reachability plot for pick-up points in peak hours can be calculated (as shown in Figure 6).

Indeed, the clusters can be represented and extracted based on the dents of the reachability plot (31, 37). However, the number of clusters (i.e., the hotspots in this paper) and the number of points contained in each hotspot are critical for the computing accuracy and computation time. In this paper, the cumulative percentage distribution of pick-ups is used to identify the best neighborhood radius. The results are shown in Figure 6.

**Figure 7:**
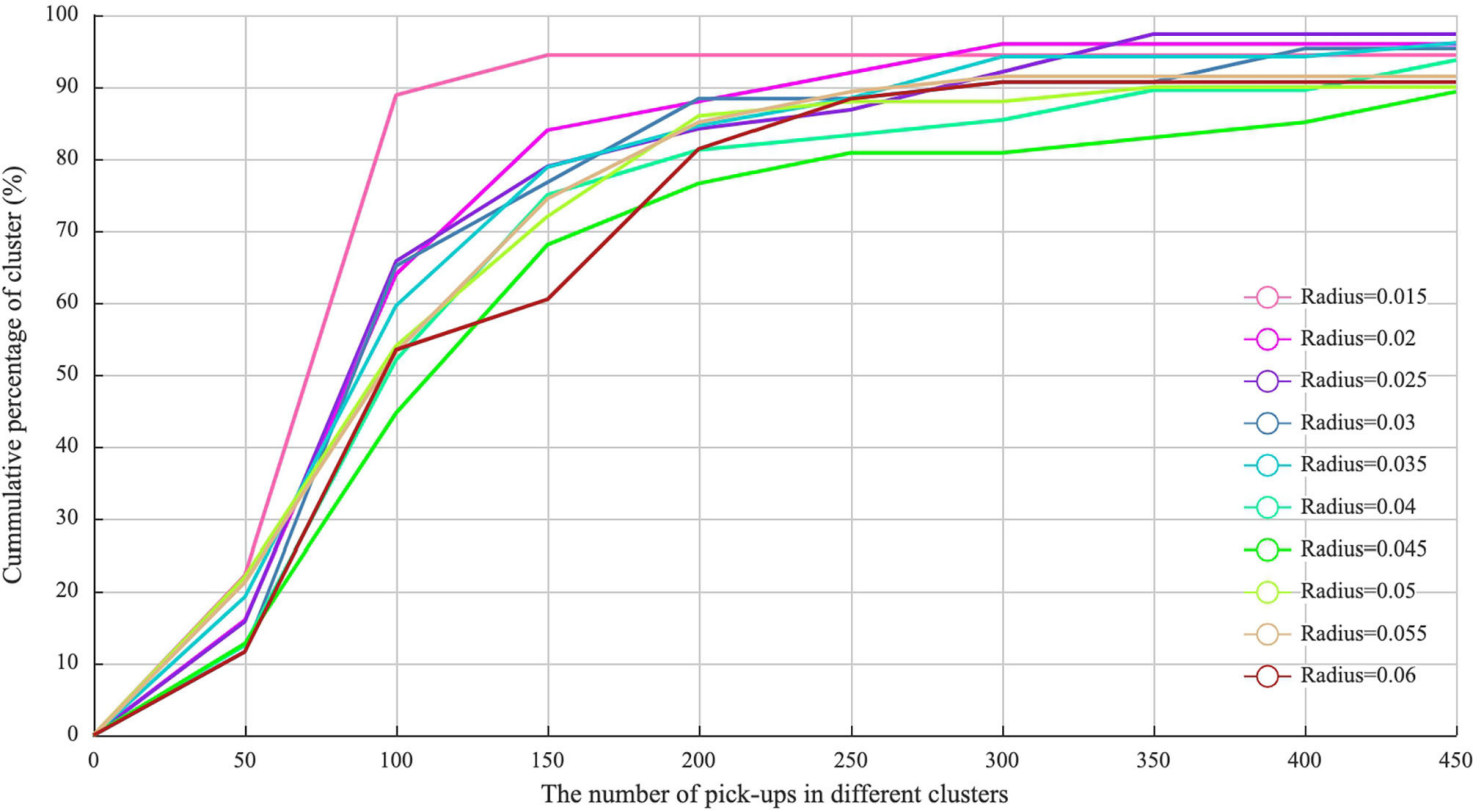
Cumulative proportion of clusters of pick-ups.

**Figure 8:**
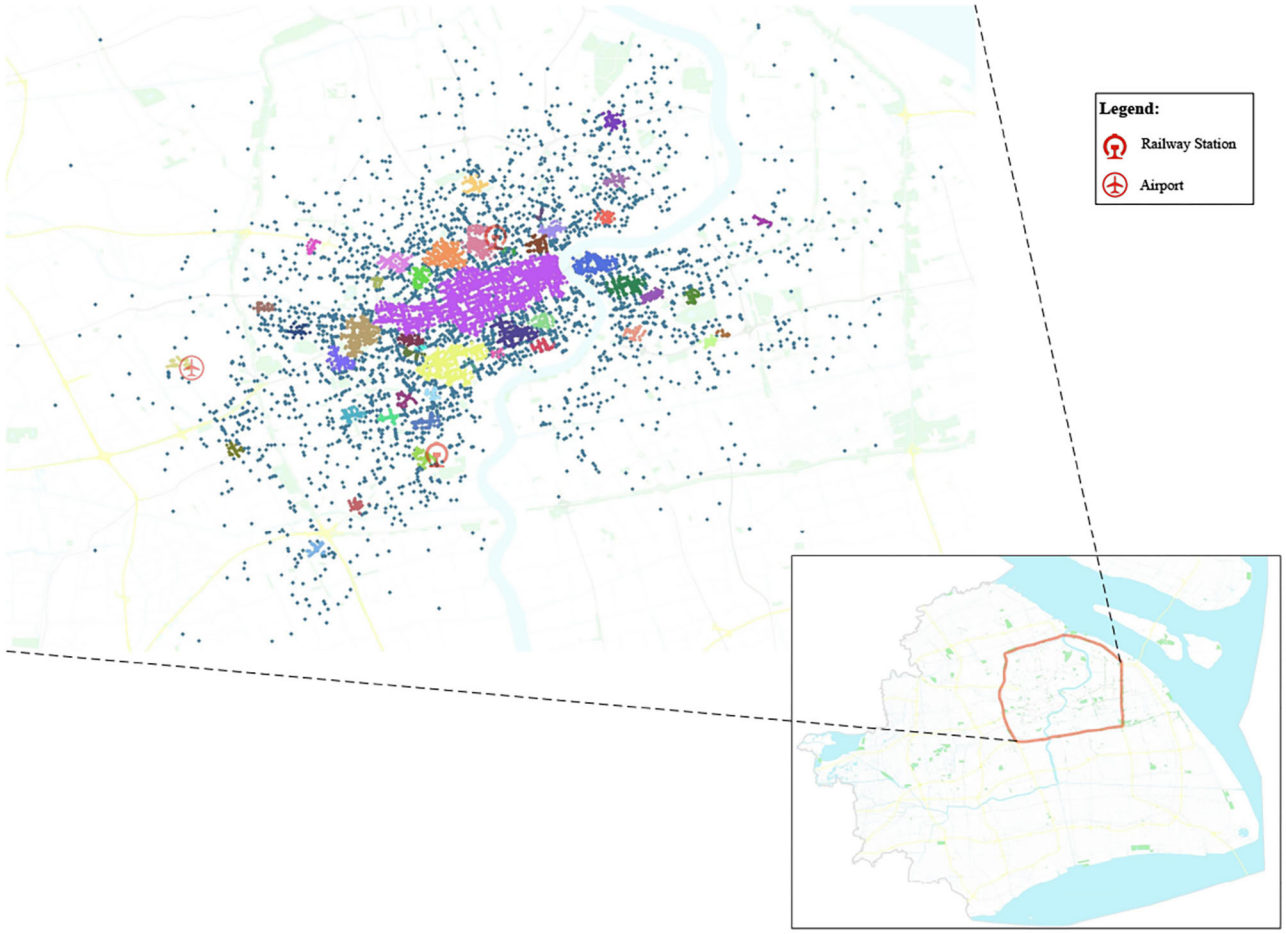
The geographical distribution of Hotspots.

As we can see in Figure 7, the number of clusters with less than or equal to 100 pick-ups accounts for 90% of the whole clusters when the neighborhood radius ε = 0.015. This indicates that many clusters with very few pick-up points are generated. Some central business districts or highly populated areas cannot be efficiently reflected. However, when ε ranges from 0.04 to 0.06, the percentage of clusters with less than 450 pick-up points reaches 90%, illustrating the most attractive hotspots in the urban area can be identified. Besides, when ε = 0.05, more than 70% of pick-ups distributed within the Inner Ring of Shanghai City are clustered. Thus, in this paper, the neighborhood radius is set as ε = 0.05. The value of the neighborhood radius is also presented in the black horizontal line of Figure 6. Based on the reachability plot and neighborhood radius, 47 hotspots are finally identified by the OPTICS algorithm and visualized in Figure 8.

### 4.3. Multicollinearity Detection

**Figure 9:**
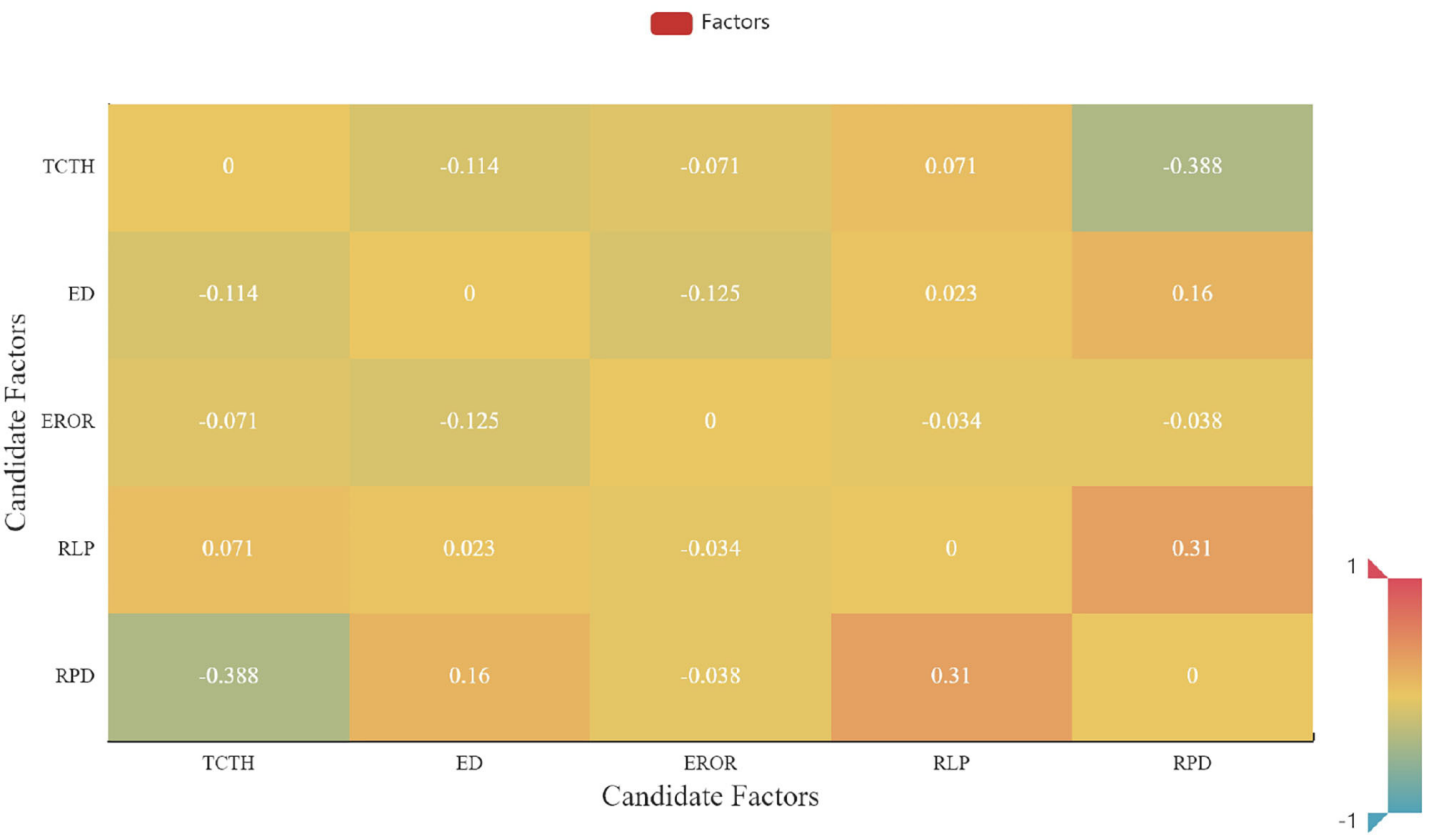
The distribution of Pearson product-moment correlation coefficient.

Before the calibration of the MNL model, multicollinearity detection is firstly conducted. In this section, the PPMCC between any two factors considered in the utility function of the MNL model for off-peak hours is calculated and shown in Figure 9. As shown in Figure 9, the maximum value among the correlation coefficients is 0.388, smaller than the threshold value of 0.7. Thus, results reveal that there is no presence of collinearity for any pairwise correlations between any two candidate factors.

To further detect the presence of multicollinearity, the VIF is also computed using Stata 14.0, and the results are presented in Table 2. The VIF values for all candidate factors, ranging from 1.027 to 1.385, are approximately equal to 1 and much less than 10. The results further verify that no collinearity is presented, and the candidate factors are reasonably selected in the utility function of the MNL model. The multicollinearity detection method for the morning-peak and evening-peak models is the same as discussed above. The full results for all periods can be found in Appendix C.

**Table 2:** The variance inflation factors.

**Candidate factor**	**VIF**	**Sqrt(VIF)**	**Tolerance**
RPD	1.385	1.18	0.722
RLP	1.162	1.08	0.861
EROR	1.027	1.01	0.974
ED	1.046	1.02	0.956
TCTH	1.251	1.12	0.799

*RPD, Relative Passenger Demand; RLP, Regional Likelihood of Pick-ups; EROR, Expected Rate of Return; ED, Enroute Delay; TCTH, Traffic Condition of Target Hotspot*.

### 4.4. Calibration Results

Using the maximum likelihood method, the coefficient of each variable in the MNL model is calibrated, and the results are shown in Table 3. First, for morning peak hours, the Cox & Snell Rho-squared and Nagelkerke Rho-squared of the MNL model are 0.525 and 0.706, respectively. The Cox & Snell Rho-squared and Nagelkerke Rho-squared of the MNL model for off-peak hours are 0.429 and 0.55. Moreover, the values for the evening peak hours' model are 0.24 and 0.32. The values of the two parameters demonstrate that the models have a good fit.

**Table 3:** Estimation Results of the time-dependent MNL model.

**Factors**	**Coefficient**^*****a*****^ **[Std.Err]**^*****b*****^		
	**Morning-peak**	**Off-peak**	**Evening-peak**
	**hours**	**hours**	**hours**
Constant	–5.536 [0.335]	4.619 [0.321]	2.422 [0.262]
EROR	2.83 [0.12]	2.435 [0.096]	2.338 [0.073]
RPD	3.549 [0.24]	2.219 [0.0301]	3.421 [0.273]
TCTH	–0.217 [0.013]	–0.368 [0.015]	–0.122 [0.012]
RLP	16.802 [0.405]	3.405 [0.558]	3.926 [0.251]
ED	–0.000255	–0.001	–0.000107
	[0.000014]	[0.000086]	[0.000022]
Number of observations	25,923	22,507	16,864
–2 Log likelihood	5324.984	6028.273	9220.762
Cox&Snell R Square	0.525	0.429	0.240
Nagelkerke R Square	0.706	0.550	0.320

a *All parameters are at a 99% confidence level*.

b *The values in bracket represent the standard errors of the candidate variables*.

Second, the sign of the constant of the utility function indicates the preferences of vacant taxi drivers to make intra-hotspot circulating or cross-hotspot traveling. The negative constant in the morning peak hours' model shows that the vacant taxi drivers prefer the intra-hotspot circulating to find the next passenger. However, for the off-peak hours model and evening-peak hours model, the constants of the utility functions are both positive. This indeed concurs with the fact that due to the high travel demand in morning peak hours, vacant taxi drivers could find a nearby passenger in a short time because nearly every person needs to go to work in the morning (see Figure 5). By contrast, vacant taxi drivers may need to make cross-hotspot traveling to find the next order during the off-peak period in the face of shrinking demand. An interesting point worth pointing out is the constant of the utility function of the evening-peak period modeling. Typically, the travel demand in evening-peak hours is also relatively higher than in the off-peak period. Vacant taxi drivers should be able to find the next passenger after finishing an order quickly. However, the constant of the utility function in evening-peak hours is found to be positive in this study. This could be explained by the mismatch between the workplace and the dwelling. After delivering the passengers to their homes, vacant taxi drivers cannot find nearby passengers heading downtown. Thus, they prefer cross-hotspot traveling to find the next passenger during evening-peak hours.

Third, the model-fitting results show that all candidate factors affect customer-search behavior and are significant at the 99% level. It also proves that the proposed model is reasonable to describe the customer-search behavior of a vacant taxi driver. Besides, the coefficients of the expected rate of return, relative passenger demand, and the regional likelihood of pick-ups in all periods are positive, and the remaining two coefficients, traffic condition of target hotspot and en-route delay, are negative. All signs of the coefficients are logical because the area with a higher expected rate of return, relative passenger demand, and the regional likelihood of pick-ups attract more vacant taxi drivers to search for customers. However, the abominable traffic condition of the target hotspot and severe en-route delay suppress the customer-search willingness of taxi drivers.

Table 3 also shows that the magnitudes of the EROR for different periods are relatively close. It suggests that the perceptions of the EROR for taxi drivers are similar across the day. The results make sense because taxi drivers are always the pursuers of profit maximization. Similar to the perception of the EROR, the perception of the ED also has a minor fluctuation across periods. The coefficients of the ED are all negative, which suggests that the ED has a negative effect on the customer-search behavior in all modeling periods.

However, unlike the EROR and ED, the differences in the coefficients of RPD for different periods are more prominent. The magnitudes of the coefficients of RPD for the peak periods (i.e., the morning and evening peak hours) are more significant than that for the off-peak period. This could be explained by the relatively high travel demand during peak periods, and vacant taxi drivers could find the next customer in a relatively short time. However, for the off-peak period, taxi drivers need to travel a long distance to the hotspot with relatively high passenger demand to find the next order.

The coefficients of the TCTH for different periods are all negative, as we discussed above. However, the critical point worth pointing out is that the coefficient of the off-peak period is lower than those for the morning and evening peak hours. It indicates that vacant taxi drivers are unwilling to circulate within those hotspots with adverse traffic conditions during the off-peak period. Though the abominable traffic condition during peak periods also suppresses the customer-search willingness of taxi drivers, the effect is indeed much smaller. It may be attributed to the generally poor traffic condition in the entire urban area.

Regarding the RLP, its coefficient values range from 3.405 to 16.802, showing a fluctuation across different periods. Notably, the coefficient of the morning-peak model is much larger than those of evening-peak and off-peak models. It shows that vacant taxi drivers make a greater emphasis on the regional likelihood of pick-ups when they search for passengers during the morning peak period. Based on the Standard Error given in Table 3, it can conclude that the RLP, RPD, and EROR are the most significant factors affecting the customer-search behavior of vacant taxi drivers, followed by the importance of the TCTH and ED. Finally, to examine whether the customer-search behavior of vacant taxi driver changes across different periods, the Watson and Westin pooling test is applied, and the results are shown in Table 4.

**Table 4:** The results of the pooling test.

**Model**	**Log likelihood**					**LR ^***a***^**	**Threshold value**	**Conclusion of the hypothesis test ^***d***^**
	**Morning-peak model**	**Off-peak model**	**Evening-peak model**	**Sum**	**Combined model**			
1	–2662.492	–3014.1365	-	–5676.6285	–6234.2135	1115.17	15.086 ^*b*^	Reject
2	–2662.492	-	–4610.381	–7272.873	–8662.6525	2779.559	15.086 ^*b*^	Reject
3	-	–3014.1365	–4610.381	–7624.5175	–8010.607	772.179	15.086 ^*b*^	Reject
4	–2662.492	–3014.1365	–4610.381	–10287.0095	–11984.344	3394.669	23.209 ^*c*^	Reject

a *LR is the abbreviation of the log likelihood ratio*.

b *The threshold value represents the Chi-square critical value with 5 degrees of freedom at 99% confidence level*.

c *The threshold value represents the Chi-square critical value with 10 degrees of freedom at 99% confidence level*.

d *Null hypothesis test at 99% confidence level*.

Columns 5–6 present the log-likelihoods of the sum of individual period models and the four combined models. Based on Equation 12, the log-likelihood ratio (LR) can also be calculated and shown in column 7. For combination models 1–3, the degree of freedom is 5, and the Chi-square critical value is 15.086 at 99% confidence level. While for combination model 4, the Chi-square critical value with 10 degrees of freedom at 99% confidence level is 23.209. By comparing the values in columns 7 and 8, it can be found that the Chi-square critical values are significantly lower than the LRs. Thus, the null hypothesis that there is no difference between the individual period model and the combined model will be rejected. In other words, the customer-search behavior of vacant taxi drivers varies with the time of day, and we cannot pool the whole day data to mine the underlying patterns of the customer-search behaviors of taxi drivers. The results also implicate that the regulatory restraints for limiting taxi circulation or improving the taxi system performance should not be static but vary depending on the time of day.

Finally, some conclusions and interesting insights can be drawn from this paper, and they are consistent or inconsistent with some ideas in previous studies.

1. The regional likelihood of pick-ups is the most significant determinant affecting the customer-search behavior of a vacant taxi driver. This finding is consistent with the assumption in previous related research that vacant taxi driver prefers to travel toward areas with the highly successful probability of picking a passenger up (14, 38).
2. Previous studies found those taxi drivers are susceptible to the reliability of travel time (4, 20, 23, 39). This paper proposed two factors to portray the reliability of travel time. One is the traffic condition of the target hotspot, and the other is the en-route delay. Compared with the significance of the traffic condition of the target hotspot, the en-route delay shows a minimal effect on customer search behavior.

## 5. Conclusion

This paper intends to study the customer-search behavior of a vacant taxi driver by mining over 1.6 million GPS records from about 8,400 taxis. To achieve the goal, we first divide the Inner Ring of Shanghai City into 47 hotspots according to the pick-up points in peak hours. Then five candidate factors that may affect the customer-search behavior are defined. To identify the quantitative relations between the factors and the customer-search decision, several time-dependent MNL models are developed and calibrated. By using the Watson and Westin pooling test, the time-varying characteristics of the customer-search behavior of a vacant taxi driver are also presented. Some interesting insights and conclusions are found in this paper and summarized as follows.

1. The hotspots with high-density pick-ups could adequately reflect the customer-search decisions of vacant taxi drivers. Specifically, the number of pick-ups in the identified hotspots accounts for around 70% of the total demand in the Inner Ring of Shanghai City. This finding indicates that vacant taxi drivers prefer to circulate airports, railway stations, hospitals, large-scale shopping centers, and other densely populated locations but rarely search for their passengers in remote areas.
2. The regional likelihood of pick-ups (RLP), relative passenger demand (RPD), expected rate of return (EROR), traffic condition of target hotspot (TCTH), and en-route delay (ED) are all significant factors affecting the customer-search behavior of vacant taxi driver. However, the standard error of the ED is quite small, indicating that ED has a minimal effect on the customer-search decision of a vacant taxi driver. Based on the knowledge of experiences, vacant taxi drivers usually search passengers through the routes they are most familiar with.
3. The customer-search behavior of a vacant taxi driver varies with the time of day. The influence factors have a similar but not identical impact on the customer-search behavior of a vacant taxi driver. In particular, the traffic condition has much more effect on the search strategies during the off-peak period. However, during peak periods, the relative passenger demand is the most significant factor. The expected rate of return and en-route delay is, however, the factors vacant taxi drivers greatly emphasize on during the whole day. Finally, taxi drivers tend to be more susceptible to the regional likelihood of pick-ups.

The findings in this paper provide comprehensive insight into significant factors affecting the customer-search behavior of a vacant taxi driver. Against the background of the rapid development of autonomous vehicles, the results will also be useful for facilitating the commercial deployment of self-driving taxis in the future.

In this paper, only GPS records were used to study the customer-search behavior. However, this work could be further extended in further studies by incorporating other data sources, such as field survey data and loop detected data, to cross-validate the conclusions drawn in this study. In addition, due to the data protection regulation and availability, the data used in this manuscript are a little bit old. Future studies may break the limitation if the latest data are available. With the acquisition of these data, it is also interesting to analyze the evolution of the traditional taxi industry and the differences of influencing factors in these years. Furthermore, within the groups of taxi drivers, there is a substantial variation in weights attached to these candidate factors. However, in this study, the heterogeneity within the population is ignored. In future studies, it will be more interesting to investigate the heterogeneous effects of these factors.

## Data Availability Statement

The raw data supporting the conclusions of this article will be made available by the authors, without undue reservation.

## Author Contributions

LY: conceptualization, methodology, and writing–original draft. ZS: supervision and methodology. LJ: programming and writing–improvement. CC: data analysis and writing–review and editing. All authors contributed to the article and approved the submitted version.

## Funding

The work described in this article was supported by National Natural Science Foundation of China (U1811463). Finally, the authors gratefully acknowledge the support from China Scholarship Council (No. 201906060030).

## Conflict of Interest

The authors declare that the research was conducted in the absence of any commercial or financial relationships that could be construed as a potential conflict of interest.

## Publisher's Note

All claims expressed in this article are solely those of the authors and do not necessarily represent those of their affiliated organizations, or those of the publisher, the editors and the reviewers. Any product that may be evaluated in this article, or claim that may be made by its manufacturer, is not guaranteed or endorsed by the publisher.
