# Supplementary material for: A Data-Driven Customer-Search Modeling With the Consideration of Traffic Environment
Source: Front Public Health. 2022 Mar 17;10:848748. doi: 10.3389/fpubh.2022.848748 (PMC8993508; doi:10.3389/fpubh.2022.848748)
Supplement: Supplementary file 1 [file Table_1.pdf]

# A Data-driven Customer-search Modeling with the Consideration of Traffic Environment

Lan YU[a], Zhuo SUN[a], Lianjie JIN[a], Chao CHEN[a,b,*]

[a] *College of Transportation Engineering, Dalian Maritime University, Dalian, 116026, PR China*
[b] *State Key Laboratory of Structural Analysis of Industrial Equipment, School of Automotive Engineering, Dalian University of Technology, Dalian 116024, PR China*

## Appendix A. The procedure of the OD extraction and mapping algorithm

Before reporting the pseudo-code of the OD extraction and mapping algorithm, related symbols should be first introduced. $T = (CarID, Lon, Lat, Speed, Loaded, DateID)$ is the set of taxi GPS trajectories. $Group\,(T)$ is the procedure which groups set $T$ based on the attribute $CarID$. $AddSeq\,(G)$ adds an order attribute $Seq$ to Set $G$. $P$ is the set of pick-ups. $D$ is the set of drop-offs. $OD$ is the set of occupied trips. Then, the procedure of the OD extraction and mapping algorithm is shown as follows.

*Corresponding author
*Email address:* chenchaocc@mail.dlut.edu.cn (Chao CHEN )

**Algorithm 1** OD Extraction and Mapping Algorithm

$//\boldsymbol{Initialization}//$
Input: $T = (CarID, Lon, Lat, Speed, Loaded, DateID)$
$//\boldsymbol{ODExtraction}//$
$G \leftarrow Group\,(T)$
**For** each $CarID$ of $G$ **do**
    Sort the data in chronological order
    $S \leftarrow AddSeq\,(G)$
**EndFor**
**While** $S \neq \varnothing$ **do**
    Check in sequence every two adjacent elements $s_i \in S$ and $s_j \in S$
    **If** $s_i.CarID = s_j.CarID$ and $s_i.Seq = s_j.Seq - 1$
        **If** $s_i.Loaded = 0$ and $s_j.Loaded = 1$
            $P \leftarrow P \cup s_j$
        **Else If** $s_i.Loaded = 1$ and $s_j.Loaded = 0$
            $D \leftarrow D \cup s_i$
        **Else**
            Pass
    **End If**
    $S \leftarrow S \setminus s_i$
**End While**
Save Set $P$ and $D$
$//\boldsymbol{ODMapping}//$
Input: Set $P$ and $D$
$\bar{P} \leftarrow Group\,(P)$ and $\bar{D} \leftarrow Group\,(D)$
**For** each $CarID$ of $\bar{P}$ and $\bar{D}$ **do**
    Sort the data in chronological order
    $\widetilde{P} \leftarrow AddSeq\left(\bar{P}\right)$ and $\widetilde{D} \leftarrow AddSeq\left(\bar{D}\right)$
**End For**
**While** $\widetilde{P} \neq \varnothing$ or $\widetilde{D} \neq \varnothing$ **do**
    Check the element $p_i \in \widetilde{P}$ and $d_i \in \widetilde{D}$ with the same attribute $Seq$
    **If** $p_i.CarID = d_i.CarID$ and $p_i.Seq = d_i.Seq$
        $OD \leftarrow OD \cup (p_i, d_i)$
    **Else**
        Pass
    **End If**
    $\widetilde{P} \leftarrow \widetilde{P} \setminus p_i$ and $\widetilde{D} \leftarrow \widetilde{D} \setminus d_i$
**End While**
Save Set $OD$

## Appendix B. The trip characteristics

Table B1: The trip characteristics in different periods on a typical day

| Trip Characteristics | Mean[SD]* | | |
|---|---|---|---|
| | Morning-peak(7-10) | Evening-peak(17-19) | Off-peak(20-23) |
| *Vacant taxi trips* | | | |
| Search distance(km) | 2.63[3.73] | 2.50[3.29] | 6.23[6.22] |
| Search Time(min) | 10.19[9.77] | 10.60[10.82] | 17.66[14.31] |
| Search Cost( CNY) | 3.59[4.36] | 3.58[4.30] | 7.46[6.91] |
| *Occupied taxi trips* | | | |
| Journey Distance(km) | 6.08[5.45] | 5.41[4.58] | 5.67[4.77] |
| Journey Time(min) | 17.06[12.34] | 15.75[10.62] | 12.35[11.23] |
| Travel Fare( CNY) | 22.22[12.45] | 20.66[10.34] | 21.39[10.65] |
| Travel,Cost( CNY) | 7.40[5.98] | 6.71[5.08] | 6.12[5.33] |
| Profit( CNY) | 14.82[6.47] | 13.95[5.24] | 15.27[5.32] |
| Number of Orders | 12592.67 | 13832.5 | 2446.25 |

*SD is the abbreviation of Standard deviation

## Appendix C. The results of Pearson product-moment correlation coefficient and variance inflation factor

Table C1: The results of variance inflation factor

| Factor | VIF[Tolerance] | | |
|---|---|---|---|
| | Morning-Peak | Off-Peak | Evening-Peak |
| EROR | 1.023[0.977] | 1.027[0.974] | 1.007[0.993] |
| RPD | 1.408[0.71] | 1.385[0.722] | 1.209[0.827] |
| TCTH | 1.543[0.648] | 1.251[0.799] | 1.214[0.824] |
| RLP | 1.158[0.864] | 1.162[0.861] | 1.044[0.958] |
| ED | 1.001[0.999] | 1.046[0.956] | 1.013[0.987] |

Table C2: The results of Pearson product-moment correlation coefficient

| PPMCCs | EROR | RPD | TCTH | RLP | ED |
|---|---|---|---|---|---|
| EROR | [1,1,1] | [-0.062, -0.038**, 0.038**] | [0.022*, -0.071**, -0.056**] | [0.132**, -0.034**, -0.058**] | [-0.001, -0.125**, 0.019] |
| RPD | [-0.062**, -0.038**, 0.038**] | [1, 1, 1] | [-0.513**, -0.388**, -0.385**] | [0.132**, -0.034**, -0.058**] | [-0.005, 0.160**, -0.101**] |
| TCTH | [0.022*, -0.071**, -0.056**] | [-0.513**, -0.388**, -0.385**] | [1, 1, 1] | [-0.295**, 0.071**, 0.123**] | [-0.006, -0.114, 0.074**] |
| RLP | [0.132**, -0.034**, -0.058**] | [-0.002, 0.310**, 0.092**] | [-0.295**, 0.071**, 0.123**] | [1, 1, 1] | [0.028**, 0.023*, -0.035**] |
| ED | [-0.001, -0.125**, 0.019] | [-0.005, 0.160**, -0.101**] | [-0.006, -0.114, 0.074**] | [0.028**, 0.023*, -0.035**] | [1, 1, 1] |

Note:Values in bracket represent the Pearson product-monment correlation coefficients calculated in morning-peak period, off-peak period, and evening-peak period, respectively
